# Training pediatric health care providers in prevention of dental decay: results from a randomized controlled trial

**DOI:** 10.1186/1472-6963-7-176

**Published:** 2007-11-02

**Authors:** Gary D Slade, R Gary Rozier, Leslie P Zeldin, Peter A Margolis

**Affiliations:** 1 Australian Research Centre for Population Oral Health, Dental School, University of Adelaide, Adelaide, SA, Australia; 2 Department of Health Policy and Administration, School of Public Health, University of North Carolina, Chapel Hill, NC, USA; 3 Department of Dental Ecology, School of Dentistry, University of North Carolina, Chapel Hill, NC, USA; 4 Cincinnati Children's Hospital Medical Center, Center for Health Care Quality, Cincinnati, OH, USA

## Abstract

**Background:**

Physicians report willingness to provide preventive dental care, but optimal methods for their training and support in such procedures are not known. This study aimed to evaluate the effect of three forms of continuing medical education (CME) on provision of preventive dental services to Medicaid-enrolled children by medical personnel in primary care physician offices.

**Methods:**

Practice-based, randomized controlled trial. Setting: 1,400 pediatric and family physician practices in North Carolina providing care to an estimated 240,000 Medicaid-eligible children aged 0–3 years. Interventions: Group A practices (n = 39) received didactic training and course materials in oral health screening, referral, counseling and application of fluoride varnish. Group B practices (n = 41) received the same as Group A and were offered weekly conference calls providing advice and support. Group C practices (n = 41) received the same as Group B and were offered in-office visit providing hands-on advice and support. In all groups, physicians were reimbursed $38–$43 per preventive dental visit. Outcome measures were computed from reimbursement claims submitted to NC Division of Medical Assistance. Primary outcome measure: rate of preventive dental services provision per 100 well-child visits. Secondary outcome measure: % of practices providing 20 or more preventive dental visits.

**Results:**

121 practices were randomized, and 107 provided data for analysis. Only one half of Group B and C practices took part in conference calls or in-office visits. Using intention-to-treat analysis, rates of preventive dental visits did not differ significantly among CME groups: GroupA = 9.4, GroupB = 12.9 and GroupC = 8.5 (P = 0.32). Twenty or more preventive dental visits were provided by 38–49% of practices in the three study groups (P = 0.64).

**Conclusion:**

A relatively high proportion of medical practices appear capable of adopting these preventive dental services within a one year period regardless of the methods used to train primary health care providers.

**Trial Registration:**

ClinicalTrials.gov NCT00464009

## Background

Dental decay persists as a serious public health problem among young children, particularly those living in low-income households who are further disadvantaged in gaining access to dental care [1]. Only 22% of general dentists in North Carolina (NC) see at least 40 child Medicaid patients per year [2] and in 2001, only 14% of Medicaid-eligible children aged less than 2–3 years made a dental visit [3]. In contrast, visits to primary care physicians are the norm during children's first few years of life. Surveys of physicians suggest that they are willing to provide preventive dental care for their pediatric patients. Lewis et al [4] found that 74% of US pediatricians expressed a willingness to apply fluoride varnish, a concentrated form of sodium fluoride that is effective when used by dentists [5] and safe [6] for prevention of decay in children.

Yet little is known about the type of instruction and support that would be needed in order for primary health care providers to effectively adopt preventive dental procedures. Systematic reviews of continuing medical education (CME) conclude that it has limited effectiveness when provided in a single session of didactic instruction [7-10]. We and others have developed enhancements to traditional CME programs that provide feedback and offer in-office support for physicians and their staff as they adopt new procedures [11]. There is evidence that those additional measures, when added to didactic instruction, are effective in promoting counseling of parents in anticipatory guidance for pediatric health maintenance [12]. Similarly, we found that CME enhancements were effective in promoting physicians' adoption of immunizations and screenings guidelines [11].

The primary aim of this study was to compare the impact of three forms of CME on provision of preventive dental services to Medicaid-enrolled children within primary care physicians' offices in NC. We hypothesized that didactic instruction, alone, would be insufficient to produce meaningful rates of preventive dental services provision, but that additional in-office support offered to physicians and staff would result in provision of services at a rate that was comparable to dentists' provision of preventive dental services to Medicaid-eligible children. This expectation was based on our experience from a pilot study in which 66 NC medical practices were provided with conventional CME classes in the provision of preventive dental services. Subsequently, 890 Medicaid-eligible children received preventive dental services in the three-month period (2/16/00 to 5/26/00), equivalent to a rate of 8.7% among all Medicaid-enrolled children attending those practices during the period. However, among a subset of three pediatric practices that received additional in-office support, preventive dental services were provided to 18.3% of their patients in the target population. We judged the latter to be a "useful" rate of service provision when judged against the reported 20% of Medicaid-enrolled children who reportedly receive preventive dental services in dental offices [13].

During planning for this study, there was speculation from members of our collaborating medical societies that practice characteristics such as practice size and type of specialty would influence rates of adoption, in addition to any effects of CME. Specifically, there was a belief that practices that saw relatively small numbers of Medicaid-eligible children aged less than 3 years would be less likely to incorporate preventive dental services into their clinic routine, and therefore be less likely to provide these services. Hence, a secondary aim was to identify characteristics of physicians' practices that were associated with provision of preventive dental services.

## Methods

### Settings and locations

In January 2001, the NC Division of Medical Assistance (DMA) began a statewide program that reimbursed physicians for provision of preventive dental services comprising oral screening, referral as needed, parent/caregiver education, and fluoride varnish application. Reimbursement initially was $43 for a child's first visit where all three services were provided and $35 for subsequent visits by the same child. Reimbursement was limited to services provided to Medicaid-enrolled children aged from birth to the third birthday, with up to a maximum of six visits per child. In order for practices to receive reimbursement, all medical personnel who were to provide the preventive services (physicians, physician assistants, nurses, medical office assistants) were required to undergo a CME program accredited by the NC Pediatrics Society (NCPS) and the NC Academy of Family Physicians (NCAFP). That CME program used the didactic components of CME provided to all practices in this study (described below).

The target population of practices for this study was private pediatric and family physician practices in North Carolina that provided care to Medicaid-eligible children. Excluded from the study were forty pediatric and family medicine practices that had participated in a related pilot program and six practices that were enrolled in a separate study of oral health promotion. Based on 1999 service provision data, there were 1,400 eligible medical practices in NC, with a patient base of 240,000 Medicaid-eligible children aged 0–3 years. We intended to enroll 120 practices from among a targeted group of 160 "high-volume" practices comprising 104 pediatric practices that provided care to between 434 and 14,750 Medicaid eligible children aged 0–3 yrs in 1999, and 56 family medicine practices that provided care to between 258 and 1,720 such children in 1999. The rationale was that "high volume" practices potentially have the greatest population impact, and that they would optimize opportunities to detect an effect of enhanced CME, if it occurred. However, after the first six months of enrolment, it became necessary to open enrolment to all private pediatric and family physician practices, regardless of practice volume, in order to achieve the goal of 120 enrolled practices. Practices were recruited between January 2001 and February 2002. While neither the intended nor the actual sampling design could yield a sample of practices that was representative of all pediatric and family physician practices in NC, and hence lacked external validity, the method of random allocation meant that the study had internal validity for assessing the potential benefits of CME methods.

### Enrollment of practices

Eligible practices that signified an intention to attend a CME class were sent information about this study together with questionnaires about practice characteristics. The information package explained that CME was required for all medical personnel (physicians, physician assistants, nurses, medical office assistants) who would provide the preventive dental services, and that we wished to enroll all such personnel in the study. Practices in which at least one physician completed a questionnaire prior to CME were regarded as enrolled practices. Enrolled practices were assigned at random to one of three forms of CME described below. A stratified, block randomization plan was developed using PC-PLAN software v1.0 [14]. Blocks comprising 12 practices of pediatric offices or family medicine offices were allocated in equal numbers to the three intervention groups. As each practice was enrolled, it was allocated by the study coordinator to the next randomly-generated letter (A, B or C) within the relevant stratum (pediatric practice or family physician practice). Because it was not feasible to conceal allocation, each practice was advised in writing of its CME intervention group by the study coordinator.

### CME Interventions

Medical personnel in Group A practices took part in a 90 minute lecture with slides, case-based presentations and discussions of the clinical interventions. The training program is described in detail elsewhere [15]. The lecture included instruction in children's dental development, common dental diseases and their prevention, screening, referral, counseling and fluoride varnish application. Attendees were provided with samples sufficient to provide fluoride varnish to 10 patients, written course materials providing instruction about clinical procedures, and parent information brochures. The application of fluoride varnish was demonstrated using a plastic model of teeth. All practices that undertook CME were mailed a newsletter periodically that reinforced information given in the CME course and also provided updates about oral health.

Medical personnel in Group B practices received the same intervention as group A and additionally were offered support through telephone conference calls once every two weeks. The conference calls used methods developed for "learning collaboratives" in which staff receive ongoing support from CME instructors and learn from one another as they begin to implement systems for preventive care in their practices [11]. The conference calls were moderated by research staff with clinical expertise in primary health care who had assisted in other interventions among NC pediatric and family medicine offices.

Medical personnel in Group C received the same intervention as Group B and were offered additional in-office support for implementation of preventive dental procedures provided by a dental hygienist. She visited offices and provided technical assistance and advice as needed to all office staff. The support varied from suggestions for record keeping to clinical procedures in application of varnish. In many instances, it included "hands on" demonstration of the varnish application, in which the dental hygienist demonstrated its application on one child in the practice, and then assisted while medical personnel applied varnish to other children. We sought to be flexible in the type of support provided during in-office visits recognizing that a single style and content of training would not suit all learners and all practices.

### Outcome measures

The primary outcome measure was the rate of preventive dental services provision, computed as the number of children receiving preventive dental services divided by the number of children attending for a well-child visit. Rates were computed for the first 12 months following each practice's enrolment and for the period through June, 2003. The number of well-child visits during that period was used as denominator to compute the rate of service provision, adjusting for practice volume. A secondary outcome measure was defined as the proportion of enrolled practices that provided 20 or more preventive dental visits during the first 12 months following each practice's enrolment. This threshold was selected to permit comparison with findings from Mayer et al [2] who reported frequency of visits to dentists by Medicaid-eligible children.

### Sources of data

Outcome measures were computed using reimbursement claims data submitted to the DMA and provided to the investigators covering periods of three months. The numerator was a count of the number of visits where reimbursement was provided for preventive dental preventive services. Because a single child's services could not be reimbursed more than once during a three-month period, this numerator number of visits was equivalent to the number of children who received preventive dental services. A second file provided by DMA contained the number of well-child visits by children aged less than three years for whom any medical services were reimbursed, and it was used to calculate the denominator for rates. Additional information about practices obtained from the same file included: the type of practice (pediatric or family physician); whether or not the practice was one of the 160 practices targeted for recruitment, and, among those targeted practices, the number of Medicaid-enrolled patients seen in 1999, as recorded in the sampling frame. Practices were also classified according to the number of well-child visits they provided during the study period, dichotomized as fewer than 825 well-child visits/year versus 825 or more well-child visits per year. The two files were linked for analysis using the practice's unique identification code.

### Statistical analysis

Consistent with CONSORT requirements for analysis of randomized controlled trials, we undertook statistical evaluation using "intention-to-treat" analysis, where outcomes were evaluated among all randomized practices, regardless of their uptake of CME interventions. The rate of preventive dental service provision was computed for each practice by summing separately the numerator number of preventive dental visits in each quarter and the denominator number of well-child visits in each quarter and computing the quotient. To evaluate our primary aim, rates of preventive dental service provision for each CME group were compared using data from all quarters from practice enrollment through June 2003 and for only the first four quarters (i.e. 12 months) following enrolment. Rates were compared statistically using Poisson regression. In order to adjust for over-dispersion, we adjusted all standard errors and test statistics by a scale parameter equal to the square root of the observed deviance of the model divided by its degrees of freedom [16]. The probability of practices providing at least 20 dental preventive services within 12 months after enrolment was also computed to form the secondary outcome variable and compared using the Chi-square test. Further comparisons among CME groups were undertaken with stratification by enrollment phase (i.e. whether or not practices were in the targeted enrolment group) and type of specialty (pediatric or family physician).

Our secondary aim was evaluated by comparing rates among practices classified by specialty and practice volume, the latter indexed by the practice's summed denominator value for the first 12 months after CME, and dichotomized at the median to classify practices as low-, and high-volume practices. We additionally described, but did not statistically evaluate, rates of preventive dental service provision through June 2003 using a "per-protocol" analysis. This analysis was achieved by computing rates separately for practices that did and did-not participate in the additional CME interventions of conference calls (groups B and C) and in-office visits (group C).

### Sample size

We made projections about expected effect sizes using data from our pilot study of 66 NC medical practices that were provided with conventional CME classes following which 8.7% of Medicaid-enrolled children attending those practices received preventive dental services. In contrast, the rate was 18.3% among children attending practices that had received additional in-office support. Based on these data, we calculated that enrolment of 84 high-volume practices would yield 72,273 visits by children in the target age group during the first year. That number would be sufficient to detect an absolute difference as small as 4% in rates of service provision among the three interventions using a two-tailed Type I error of P = 0.016 (equivalent to P = 0.05 for each of three pairwise comparisons using Bonferroni's correction for multiple comparisons), power of 0.80; and design effect = 2.0 to increase variance accounting for clustering of children (who are units of analysis for this primary outcome) within practices (which are units of randomization). Following funding of the project, the NC Academy of Family Physicians expressed a wish to have additional family medicine practices included in the study, so the enrolment procedures was stratified to specifically target 36 such practices. A post-hoc calculation based on the preceding assumptions about effect size, TypeI error and design effect yielded an increase in power to 95% associated with the increase in sample size.

### Ethical conduct of research

All medical personnel signed informed consent to participate in the study. The study was reviewed and approved by the UNC School of Dentistry's Committee on Investigations Involving Human Subjects.

## Results

**Figure 1:**
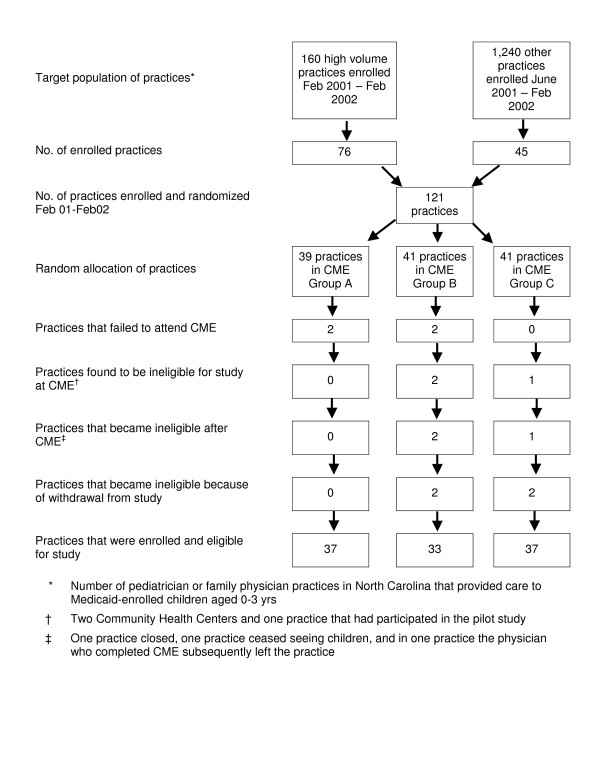
Enrolment and retention of medical practices.

A total of 121 practices were enrolled and allocated at random to one of the three study groups over the full 13 month enrollment period (Fig 1). Seventy six of the enrolled practices were from the original group of 160 practices targeted for enrolment, and the remaining 45 were from the open enrollment period. Despite efforts to verify each practice's eligibility for the study and the willingness of its physicians to participate prior to allocation, it was necessary to exclude 14 practices from analyses either because physicians failed to attend a CME course, the practice was found to be ineligible for the study at the time of the CME course, practices subsequently became ineligible or they withdrew from the study (Fig 1). Hence, data for analysis of adoption rates were available from the remaining 107 eligible practices that were enrolled in and eligible for the study.

Training was provided to 323 medical personnel (171 physicians and 152 other staff) at the 107 study practices, ranging from 1 to 17 personnel per practice (1 to 7 physicians per practice), with a median of 2 personnel (1 physician) per practice. A single person was trained at 43 practices, two people were trained at 27 practices, while at the remaining 37 practices, three or more people were trained. There was no statistically significant difference among study groups in the number of trained personnel per practice (P = 0.40) or trained physicians per practice (P = 0.97, Kruskal-Wallis test).

Larger practice volume, as indexed by the number of Medicaid-enrolled children aged 0–3 years in 1999, was associated with a greater probability of being enrolled and eligible for the study (Table 1). However, no statistically significant differences in the probability of enrollment and eligibility were found between pediatric and family physician practices. As expected under random allocation, the percentage of practices that were pediatric offices (57–62%, P = 0.88) and that were targeted for enrollment (54–72%, P = 0.27) did not differ significantly among CME groups. Additionally, practice volume, as indexed by the median number of well-child visits by children aged 0–3 years in the first 12 months after enrollment, ranged from 717 to 1024, but did not differ significantly (P = 0.96) among CME groups.

**Table 1:** Characteristics of practices associated with study participation among 160 targeted medical practices

Practice characteristic	No. of practices targeted	% of targeted practices enrolled	P-value	% of targeted practices enrolled & eligible	P-value
Practice volume					
<500 patients*	48	37.5		27.1	
500–<1000 patients	60	48.3	0.19^†^	45.0	0.03^†^
≥1000 patients	52	55.8		51.9	
Specialty					
Family medicine	55	43.6	0.51^‡^	34.5	0.18^‡^
Pediatrician	105	49.5		45.7	

All targeted practices	160	47.5		41.9	

* No. of Medicaid enrolled patients aged 0–3 yrs seen in 1999

† P-values are from Chi-square test (2 df)

‡ P-value is from Chi-square test (1 df)

Only about one half of practices in Groups B and C participated in additional CME interventions, despite them being promoted heavily by research staff. Ten of the 33 practices in Group B and 12 of the 37 practices in Group C participated in at least one conference call; 24 of the 37 practices in Group C had an in-office visit.

**Figure 2:**
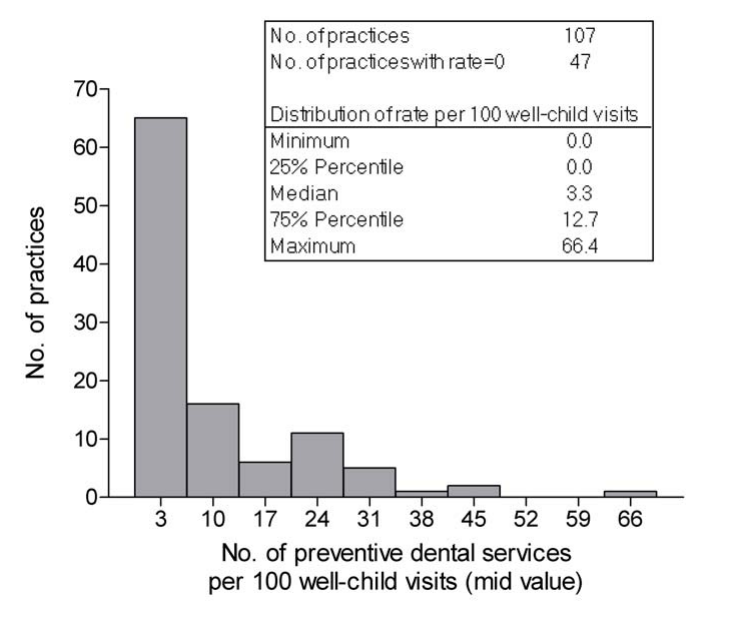
Histogram of rate of preventive dental services provision per 100 well-child visits.

During the first 12 months following enrollment, 107 practices provided a median of 824 well-child visits for children aged 0–3 years (range = 0 – 12,860 visits) with 104 practices providing one or more such visits. During that period, 56% of practices provided at least one preventive dental visit, 43% provided at least 20 such visits, and 36% provided at least 40 such visits. For the period of full claims enumeration, through June 2003, all practices provided at least one well-child visit for children aged 0–3 years (median = 1,711, range = 33 – 19,558 visits) and 63% of practices provided at least one preventive dental visit. The overall rate of service provision through June 2003 was 10.0 preventive dental services per 100 well-child visits, although the rate varied considerably from 0 in 47 practices, to 66.4 in one practice. This produced a skewed distribution of rates among the 107 practices (Fig 2).

There were small and statistically non-significant differences among CME groups in rates of preventive dental services provision, whether measured within the first 12 months after enrollment, or using all available service provision data through June 2003 (Table 2). For example, during the first 12 months after enrollment, the service provision rate in Group B (12.9 preventive dental services per 100 well child visit) was approximately 25% greater than GroupC (8.5 preventive dental services per 100 well child visit), while Group A rates fell within that range. However, 95% confidence intervals overlapped substantially, and the corresponding P-value of 0.32 did not approach the threshold for statistically significance (Table 2). While rates of service provision were marginally higher using all available claims data through June 2003, only small and statistically non-significant differences were observed among CME groups. Similarly, the probability of practices providing at least 20 preventive dental services did not differ among CME groups (Table 2). Although not shown in Table 2, among the 46 practices that provided 20 or more preventive dental services in the first 12 months, the rate of preventive dental visits was 16.2 (95%CI = 13.0 – 20.2) per 100 well-child visits during the first 12 months following enrolment.

**Table 2:** Probability of adoption and rates of preventive dental visits among CME groups

		Adoption during first 12 months after enrolment		Adoption through June 2003
			
CME group*	No. of practices	% of practices providing ≥ 20 preventive dental visits	No. of preventive dental visits per 100 well-child visits (95% CI)	No. of preventive dental visits per 100 well-child visits
A	37	48.6	9.4 (6.1–14.4)	11.6 (8.0–16.8)
B	33	42.4	12.9 (8.7–19.0)	15.2 (10.8–21.4)
C	37	37.8	8.5 (5.6–12.8)	9.9 (6.8–14.4)
All practices	107	43.0	10.0 (7.9–12.0)	12.0 (9.7–14.8)
*P-value*		*0.64*^†^	*0.32*^‡^	*0.24*^‡^

* CME (continuing medical education) group A = didactic instruction;

CME group B = didactic instruction plus conference calls;

CME group C = didactic instruction plus conference calls plus in-office support

† P-value is from Chi-square test (2 df)

‡ P-values are from Poisson regression likelihood ratio Chi-square (2 df)

**Figure 3:**
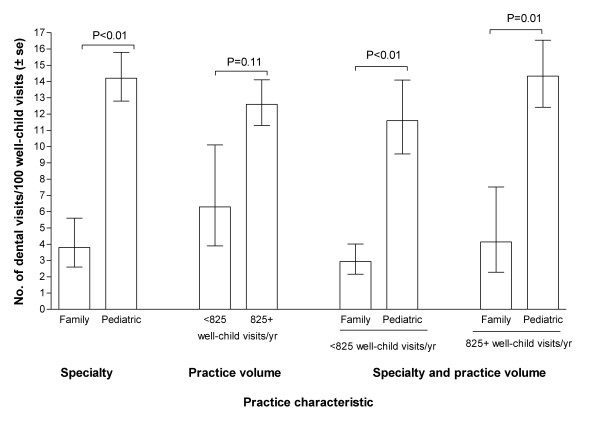
Rates of dental preventive service provision through June 2003 among medical practices classified by specialty and practice volume.

In evaluating the second aim, we found that two practice characteristics were important predictors of preventive dental service provision: type of specialty and practice volume (Fig 3). Using all available data through June 2003, the rate was three times greater among pediatric practices (14.2 preventive dental visits per 100 well-child visits) than among family medicine practices (3.8 preventive dental visits per 100 well-child visits, P < 0.01 – Fig 3). While high practice volume (above the median of 825 well child visits during the first year after enrolment) was associated with a two-fold difference in rates of preventive dental services provision, it was not statistically significant (P = 0.11, Fig 3). Practice volume varied substantially between family medicine practices (median = 314 well child visits in the first year after enrollment) compared with pediatric practices (median = 1468 well child visits – data not shown). However, as illustrated in Fig 3, within each stratum of practice volume, pediatric practices persisted with a 3- to 4-fold greater rate of preventive dental service provision compared with family physician offices. Nonetheless, adjustment for type of specialty and practice volume did not alter the statistically non-significant differences among study groups in Table 2 (results not tabulated).

When group B practices were analyzed "per protocol", the rate of preventive dental visits through June 2003 was 21.5 services per 100 well child visits among the subgroup of 10 practices that participated in one or more conference calls compared with 3.9 services per 100 well child visits among the 23 practices that did not participate in conference calls. Within group C practices, the rate was 11.7 services per 100 well child visits for the 24 practices where an in-office visit was made compared with 0.1 services per 100 well child visits for the 13 practices where no in-office visit was made.

## Discussion

The main finding from this study was that rates of preventive dental services provided in medical offices to young, Medicaid-enrolled children were not influenced by the type of CME offered to physicians and their staff. This was a surprising result, first because traditional CME, as offered to GroupA practices in 90 minute didactic sessions, generally is thought to be ineffective in producing change in practice behavior [9]. Yet even among GroupA practices in this study, nearly one half of practices (48.6% – Table 2) provided at least 20 preventive services during the year following CME and nearly 10% of Medicaid enrolled children making well-child visits to those offices received the preventive services (9.4 services/100 well child visits – Table 2).

One explanation for this null result is that only about one half of practices in CME groups B and C participated in the additional CME interventions that were offered. Yet, when developing this project, we felt that support in addition to traditional CME would be critical because most physicians receive little instruction in oral health care during their training [17] and one aspect of the intervention, fluoride varnish, had become available only recently in the US [6]. For that reason, we dedicated the time of one project staff member to providing CME, including in-office visits to provide hands-on assistance, and her efforts were supported by the professional societies that promoted the intervention, together with our own research staff who had considerable experience with implementing change in medical practices. The null effect was all the more surprising in view of qualitative findings from a study at one university-affiliated hospital pediatric clinic, where medical personnel were trained and supported in provision of preventive dental services including varnish [18]. In that study, the importance of identifying a "clinical leader" and a tracking system were reported to be critical enablers of adoption, and assistance in those aspects of medical office organization frequently was provided during in-office visits.

While it is tempting to conclude that those additional CME efforts were beneficial, based on the per protocol analysis where rates of service provision were at least twice as high among practices that made conference calls and that had in-office visits, these represent discretionary and post-randomization decisions. For those reasons, the observed association between additional CME and service provision may not be causal, and hence these findings from per protocol analysis do not demonstrate effectiveness. For example, it is possible that participation in additional CME activities was merely a marker of willingness to engage in the overall intervention, while the decision not to participate may have reflected a general reluctance, unwillingness or inability to adopt these dental preventive procedures.

Other factors may explain the observed lack of difference in adoption rates between the three experimental groups in this study. An important aspect of the program was financial reimbursement ($43 for a child's first visit, $38 per subsequent visit) which was reported by several physicians to be a significant incentive. It seems likely that the opportunity for new income provided through this dental program was sufficient motivation for practitioners to contemplate providing the services. Furthermore, many medical providers said that they found the technical advice to be straightforward. These reports are consistent with our finding that pediatricians and family physicians already had quite high levels of knowledge in basic aspects of dental decay [19]. Similarly, a large majority (83%) of pediatricians in Washington State said that they already provided anticipatory guidance in oral health, prior to receiving training in dental care [20]. We note, also, that other intensive programs promoting practice change have not necessarily been effective in changing practice-based or patient-based outcomes [21].

Additional aspects of the intervention may have limited the impact of enhanced CME methods on the outcomes studied here. Unlike some "learning collaborative" interventions, where practices are enrolled only after expressing and demonstrating enthusiasm to be involved [11], we recruited widely among practices with the intention of maximizing coverage of Medicaid-eligible children in NC. Additionally, some of the enhanced CME activities were under-utilized. For example, conference calls were joined usually by only half a dozen practices, and only 25 of the 41 practices in GroupC agreed to an in-office visit.

An additional explanation for the observed lack of difference among CME groups may be due to the nature of practices that chose to enroll during the first year of the statewide program. These early-enrolling practices may have been particularly enthusiastic, thereby minimizing the potential for different forms to CME to have an effect on adoption. Lewis et al found that primary care physicians who undertook training in similar preventive dental procedures had characteristics consistent with early-adopters of innovation, including greater empathy and a more favorable attitude to change [22]. It remains possible, therefore, that more intensive CME would be influential among later-trained practices. Finally, the relatively short-term duration of follow-up reported here and the restriction of this analysis to process-based outcomes of service provision limit the capacity to comment on potential long-term changes and impact on children's oral health.

Notwithstanding the overall lack of differences among study groups, the finding that 38–49% of practices provided 20 or more preventive dental visits within 12 months represents a substantially greater probability of adoption compared with the 29% of dentists who provided dental visits to at least 20 Medicaid-eligible children per year in the period [2]. While an average of only 10.0 preventive dental services were provided per 100 well-child visits, we believe this is a substantial contribution to oral health for the NC child Medicaid population for two reasons. First, at a population level, it almost doubles the estimated rate of 20% of Medicaid-enrolled children who receive preventive dental services [13] and the 14% of 2–3 year old Medicaid-eligible children who make a dental visit [3]. Furthermore, primary care physicians are distributed widely throughout NC, in contrast to dentists: 79 of the state's 100 counties qualify as federally recognized dental professional shortage areas.

Second, at the practice level, the rate of 10 preventive dental services per 100 well-child visits is meaningful, given that the denominator count of well-child visits is represented by many visits that occur in the earliest months of life, prior to tooth eruption, while far fewer are well-child visits occurring towards the end of the age-range covered by this program. While the NC DMA preventive dental program included children up to the third birthday, the program required that all three components be provided in order for reimbursement to be claimed: screening, education and varnish application. Because teeth generally begin to erupt at the age of six months, the services effectively cannot be provided before that age, by which time the first four well-child visits scheduled in the Early Periodic Screening, Detection and Treatment (EPSDT) program would have been completed. The next six scheduled EPSDT well-child visits, occurring from 6 months through 24 months of age, therefore represented the main opportunities for medical personnel to provide these preventive dental services, although additional opportunities existed at visits other than well-child visits. The consequence is that the denominator used here to calculate rates gives a pessimistic estimate of practice activity, and the true rate could be higher, perhaps by as much as a factor of two. If that were the case, the rate in this study could arguably be doubled, to 20%, which would be comparable to the rate of 131 applications per 600 well-child visits (22%) reported in a study undertaken in one university-affiliated hospital pediatric clinic [18].

This study confirmed our expectation that large volume practices and pediatrician's offices were the settings most likely to implement these dental preventive practices. The stratified analysis (Fig 3) further suggests that the relatively higher rate of preventive dental services in pediatrician's offices is not merely a consequence of their larger practice volume compared with family physician practices. There are probably several aspects of pediatric practice, aside from practice volume, that increases the likelihood of adopting these procedures, including existing appointment systems that can readily integrate dental services into well-child visits.

## Conclusion

The key implication of this study is that, regardless of the method used for CME, a relatively high proportion of practices enrolled in this study appear capable of adopting these preventive dental services, resulting in provision of preventive dental visits to an average of 10% of Medicaid-eligible children. These represent substantial expansion in availability of preventive dental services above the population coverage currently achieved through dental offices. Importantly, these levels of adoption were achieved among practices that were among the first to seek training in this statewide program that reimbursed practitioners $38–$43 per preventive dental service – two factors that we believe may have accounted for a lack of effect of more intensive CME.

## Competing interests

The author(s) declare that they have no competing interests.

## Authors' contributions

GS contributed to the study design, served on a project oversight committee, participated in physician training, conducted the analysis, and wrote some sections of the manuscript. GR project Principal Investigator, conceived of the study, contributed to the study design, served on a project oversight committee, participated in physician training, and wrote some sections of the manuscript. LZ was project coordinator and contributed to revisions of the manuscript. PM designed continuing medical education program and contributed to revisions of the manuscript. All authors read and approved the final manuscript

## Pre-publication history

The pre-publication history for this paper can be accessed here:


